# Progressive alterations in white matter microstructure across the timecourse of Huntington's disease

**DOI:** 10.1002/brb3.2940

**Published:** 2023-03-14

**Authors:** Carlos Estevez‐Fraga, Michael S. Elmalem, Marina Papoutsi, Alexandra Durr, Elin M. Rees, Nicola Z. Hobbs, Raymund A. C. Roos, Bernhard Landwehrmeyer, Blair R. Leavitt, Douglas R. Langbehn, Rachael I. Scahill, Geraint Rees, Sarah J. Tabrizi, Sarah Gregory

**Affiliations:** ^1^ Huntington's Disease Centre UCL Queen Square Institute of Neurology, University College London London UK; ^2^ Sorbonne Université, Paris Brain Institute (ICM), AP‐HP, Inserm, CNRS Pitié‐Salpêtrière University Hospital Paris France; ^3^ Ixico London UK; ^4^ Department of Neurology Leiden University Medical Centre Leiden The Netherlands; ^5^ Department of Neurology University of Ulm Ulm Germany; ^6^ Centre for Huntington's Disease at UBC Hospital, Department of Medical Genetics and Division of Neurology, Department of Medicine University of British Columbia Vancouver BC Canada; ^7^ Department of Psychiatry University of Iowa Iowa City Iowa USA; ^8^ Wellcome Centre for Human Neuroimaging, UCL Queen Square Institute of Neurology University College London London UK

**Keywords:** diffusion tensor imaging, Huntington's disease, longitudinal, presymptomatic, symptomatic

## Abstract

**Background:**

Whole‐brain longitudinal diffusion studies are crucial to examine changes in structural connectivity in neurodegeneration. Here, we investigated the longitudinal alterations in white matter (WM) microstructure across the timecourse of Huntington's disease (HD).

**Methods:**

We examined changes in WM microstructure from premanifest to early manifest disease, using data from two cohorts with different disease burden. The TrackOn‐HD study included 67 controls, 67 premanifest, and 10 early manifest HD (baseline and 24‐month data); the PADDINGTON study included 33 controls and 49 early manifest HD (baseline and 15‐month data). Longitudinal changes in fractional anisotropy (FA), mean diffusivity (MD), axial diffusivity, and radial diffusivity from baseline to last study visit were investigated for each cohort using tract‐based spatial statistics. An optimized pipeline was employed to generate participant‐specific templates to which diffusion tensor imaging maps were registered and change maps were calculated. We examined longitudinal differences between HD expansion‐carriers and controls, and correlations with clinical scores, including the composite UHDRS (cUHDRS).

**Results:**

HD expansion‐carriers from TrackOn‐HD, with lower disease burden, showed a significant longitudinal decline in FA in the left superior longitudinal fasciculus and an increase in MD across subcortical WM tracts compared to controls, while in manifest HD participants from PADDINGTON, there were significant widespread longitudinal increases in diffusivity compared to controls. Baseline scores in clinical scales including the cUHDRS predicted WM microstructural change in HD expansion‐carriers.

**Conclusion:**

The present study showed significant longitudinal changes in WM microstructure across the HD timecourse. Changes were evident in larger WM areas and across more metrics as the disease advanced, suggesting a progressive alteration of WM microstructure with disease evolution.

## Graphical abstract

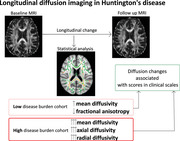

## 1 INTRODUCTION

Loss of white matter (WM) organization is a key feature of Huntington's disease (HD), with myelin thinning, reduced expression of myelin‐related genes, andincreased density of oligodendrocytes in the tail of the caudate nucleus occurring early on (Gómez‐Tortosa et al., 2001; Teo et al., 2016; Xiang et al., 2011). Morphological magnetic resonance imaging (MRI) studies provide clear evidence of progressive striatal atrophy that rapidly extends to WM during premanifest (pre‐HD) stages (Tabrizi et al., 2012, 2011, 2013). However, investigation at the microstructural level can help characterize the changes in WM organization and their relationship to the HD phenotype.

Diffusion tensor imaging (DTI) is commonly used to infer the coherence of WM tracts and WM organization in vivo. Although biological properties of brain tissue, such as the presence of crossing fibers, limit their interpretation, DTI metrics remain the most widely used method to characterize WM microstructure (Alexander et al., 2007). Cross‐sectional studies using DTI have provided robust evidence of disorganization in deep and superficial WM (Casella et al., 2020; Filippi & Agosta, 2016; Phillips et al., 2014; Wu et al., 2017) and the corpus callosum (Di Paola et al., 2012; Rosas et al., 2010) in HD many years before symptom onset (Liu et al., 2016). Cross‐sectional analyses of diffusion metrics have also shown correlations with motor, cognitive, and functional scales (Estevez‐Fraga, Scahill, Rees et al., 2021).

The composite UHDRS (cUHDRS) is a multidimensional measure of progression in HD encompassing functional, cognitive, and motor subscales that has been used as a primary outcome in the phase 3 clinical trial with tominersen, an antisense oligonucleotide targeting *HTT* mRNA. A recent study demonstrated a significant correlation between the cUHDRS and diffusivity metrics (Estevez‐Fraga et al., 2021).

An increasing number of clinical trials in HD are using diffusion imaging measures as biomarkers for drug efficacy and safety, as well as to understand the mechanisms underlying treatment response (Tae et al., 2018). Therefore, longitudinal investigation of WM microstructure is paramount. Apart from correlations with clinical function, investigating the exact point at which a certain biomarker is sensitive and determining the longitudinal sensitivity over short periods of time are crucial to defining the role of diffusion imaging metrics in clinical trials.

Unfortunately, only a limited number of studies have evaluated *change* in WM microstructure over time, with findings generally mixed (Gregory et al., 2015; Hobbs et al., 2015; Poudel et al., 2015; Sritharan et al., 2010; Vandenberghe et al., 2009; Weaver et al., 2009). However, evidencing significant longitudinal change over a clinical trial period is a necessary step toward showing change in the natural trajectory of a biomarker in response to a therapy.

It is likely that due to the methodological limitations analyzing multiple time points of diffusion data, the subtle changes that can occur in a slowly progressive degenerative disease over time may remain undetected. In the current study, therefore, we have employed a longitudinal pipeline that seeks to reduce misalignment between multiple time point scans by creating individual participant templates to which diffusion data from multiple visits are registered; this then ensures that the location of voxels is consistent across the data (Engvig et al., 2012).

We investigated WM microstructural change over the trajectory of HD, analyzing longitudinal diffusion data from two HD cohorts with different disease burden: TrackOn‐HD, composed mainly of premanifest HD (pre‐HD) and controls, with change over 24 months; and PADDINGTON, including early manifest HD and controls, with change over 15 months. For each cohort, we used the novel longitudinal pipeline in conjunction with whole‐brain voxelwise tract‐based spatial statistics (TBSS) (S. M. Smith et al., 2006) to compare change in diffusion metrics between HD expansion‐carriers and controls. We also correlated baseline scores in clinical measures with change in diffusion metrics to test the predictive ability of these scales for microstructural degeneration. We hypothesized that HD expansion‐carriers would display evidence of progressive WM disorganization from pre‐HD to manifest HD, coupled with a clear correlation between baseline function and WM degeneration.

## 2 MATERIALS AND METHODS

### 2.1 Participants

Participants with DWI data for baseline and final (third) visits were recruited from the TrackOn‐HD (time interval 24 months) and PADDINGTON (time interval 15 months) cohorts (Hobbs et al., 2013; Klöppel et al., 2015).

### 2.2 TrackOn‐HD

The TrackOn‐HD cohort recruited 239 participants (106 pre‐HD, 22 early HD, and 111 controls), from four study sites (London, Leiden, Paris, and Vancouver) evaluated annually over a period of 2 years. Gene expansion carriers were required to have ≥40 CAG repeats in the *HTT* gene and a disease burden score >250 (Penney et al., 1997). Control participants were gene‐negative volunteers and family members. At each visit, participants underwent clinical testing, a neuropsychological battery, and an MRI scanning session. Inclusion criteria required age between 18 and 65 years free from major neurological, medical, or psychiatric disorders and able to tolerate MRI. The study was approved by the local ethics committee and all participants provided written informed consent. For further details, see Kloppel et al. (2015).

We excluded 95 participants due to incomplete DWI data, incomplete clinical data, or failed quality control, while further five participants had to be excluded because of failed longitudinal registration, leaving a total of 144 participants in this study (10 early HD, 67 pre‐HD and 67 controls) (Table 1, Figure S1). There were no statistically significant differences between included/excluded participants.

**TABLE 1:** Baseline demographics of participants

	TrackOn‐HD		PADDINGTON		*p* Value
	Controls	HD expansion‐carriers	Controls	HD expansion‐carriers	
*n*	67	77	33	49	
Age	49.55 (9.84)	44.03 (8.28)	51.99 (9.13)	47.75 (9.75)	*p* < .0001
Sex (M:F)	40:27	39:38	14:19	20:29	*p* = .1758
CAG	N/A	43.12 (2.26)	N/A	43.61 (2.99)	*p* = .2983
DBS	N/A	311.32 (55)	N/A	366.12 (83.20)	<.0001
Study site	Leiden: 11	Leiden: 12	Leiden: 6	Leiden: 11	N/A
	London: 23	London: 27	London: 10	London: 13	
	Paris: 22	Paris: 24	Paris: 9	Paris:10	
	Vancouver: 11	Vancouver: 14	Ulm: 8	Ulm: 15	
Stage	N/A	pre‐HD: 67	N/A	pre‐HD: 0	*p* < .0001
		HD: 10		HD: 49	
					
Baseline TMS	1.33 (1.59)	7.43 (5.8)	1.5 (1.95)	18.71 (9.18)	*p* < .0001
Baseline TFC	13.00 (0)	12.86 (0.42)	12.97 (0.17)	12.04 (0.81)	*p* < .0001
Baseline SDMT	56.88 (11.17)	52.62 (12.00)	54.00 (9.34)	37.53 (10.41)	*p* < .0001
Baseline SWR	107.94 (17.60)	102.60 (17.16)	109.39 (14.54)	79.26 (15.21)	*p* < .0001
Baseline cUHDRS	17.87 (1.61)	16.74 (1.84)	17.67 (1.26)	13.04 (1.93)	*p* < .0001

*Note*: Values are mean +/− SD or *n* (%) group comparisons were made using one‐way ANOVA (age, SDMT, SWR, TMS, TFC and cUHDRS), *t*‐tests (CAG, DBS), and chi squared tests (sex, stage) between groups.

Abbreviations: cUHDRS, composite UHDRS; DBS, disease burden score; HD, Huntington's disease; SDMT, symbol digit modalities test; SWR, Stroop word reading; TBSS, tract‐based spatial statistics; TFC, total functional capacity; TMS, total motor score.

Given the small number of early HD participants, we combined premanifest and manifest HD participants into a single group of HD gene‐expansion carriers. We grouped together pre‐HD and manifest HD participants as imaging and histological studies have shown that pathology in HD is progressive, rather than dichotomic (Tabriz et al., 2009; Vonsattel et al., 1985). In addition, differences in disease severity between the examined cohorts is better quantified through the disease burden score (Penney et al., 1997), showing significant differences between TrackOn‐HD and PADDINGTON (Table 1).

### 2.3 PADDINGTON

The PADDINGTON cohort included 101 participants (61 early HD patients and 40 healthy controls) from four sites (Leiden, London, Paris, and Ulm) with visits at baseline, 6 months, and 15 months. HD participants were required to have ≥39 CAG repeats in the *HTT* gene and be at stage 1 of the disease, defined by a UHDRS Total Functional Capacity (TFC) ≥11. Control participants were gene‐negative partners or first‐degree family members. At each visit, participants underwent clinical testing, a neuropsychological battery, and an MRI scanning session. Inclusion criteria required age between 18 and 65 years, free from neurological, medical, or psychiatric disorders other than HD, and able to tolerate MRI. The study was approved by the local ethics committees, and all participants provided written informed consent. For further details, see Hobbs et al. (2013).

In the current study, we excluded 19 participants due to incomplete DWI, clinical data, or failed quality control, while there were no failures in longitudinal registration leaving a total of 82 participants (49 early manifest HD and 33 controls) (Table 1, Figure S2). There were no statistically significant differences in baseline characteristics between included/excluded participants.

### 2.4 Clinical scales

For our correlation analysis, we used two cognitive scales: Symbol‐Digit Modalities Test (SDMT) (Parmenter et al., 2007) and Stroop Word Reading (SWR) (Stroop, 1935), one functional scale, the TFC, and one motor scale, the UHDRS‐Total Motor Score (TMS) (Huntington Study Group, 1996). In addition, we performed correlations with the disease burden score, a function of age and CAG repeat length (disease burden score = Age × (CAG − 35.5)), which measures the amount of time that a subject has been exposed to the effects of mHTT (Penney et al., 1997) and with the cUHDRS.

The cUHDRS is a multidomain scale including the SDMT, SWR, TFC, and TMS (Equation 1), which tracks disease progression and has excellent sensitivity to disease stage (Schobel et al., 2017).

$$\begin{array}{ccc} \mathrm{cUHDRS}\; & & =\left(\frac{\mathrm{TFC}-\;10.4}{1.9}\right)-\left(\frac{\mathrm{TMS}-29.7}{14.9}\right)+\left(\frac{\mathrm{SDMT}-28.4}{11.3}\right)\\ & & +\left(\frac{\mathrm{SWR}\;-\;66.1}{20.1}\right)+10. \end{array}$$

Higher scores in all scales except for TMS and disease burden score indicate better performance.

### 2.5 MRI acquisition

Scanning protocols were standardized between sites, and inter‐scanner comparisons were performed using human volunteers or phantoms. Ixico Ltd. contributed to the development of the imaging protocol and performed initial quality control

### 2.6 TrackOn‐HD

Data were acquired on two different 3T MRI scanner systems (Philips Achieva at Leiden and Vancouver and Siemens Trio at London and Paris). T1‐weighted images were acquired using the 3D MP‐RAGE acquisition sequence. T1 imaging parameters were repetition time (TR) = 2200 ms (Siemens)/7.7 ms (Philips), echo time (TE) = 2.2 ms (Siemens)/3.5 ms (Phillips), flip angle = 10^o^ Siemens/8^o^ Phillips, field‐of‐view (FOV) = 28 cm Siemens/24 cm (Phillips), and matrix size 256 × 256 (Siemens)/224 × 224 (Phillips), yielding 208 (Siemens)/164 (Phillips) sagittal slices to cover the entire brain with a slice thickness of 1.0 mm with no gap. Diffusion‐weighted images (DWI) were acquired with 42 unique gradient directions (*b* = 1000 s/mm^2^). Seven images with no diffusion weighting (*b* = 0 s/mm^2^) or one image with no diffusion weighting were collected from the Siemens and Philips scanners, respectively. For further details, see Kloppel et al. (2015)

### 2.7 PADDINGTON

Data were acquired on four different 3T MRI scanners. T1‐weighted images were acquired using the 3D MP‐RAGE acquisition sequence. T1 imaging parameters were TR = 2200 ms, TE = 2.2 ms, FA = 10°, FOV = 28 cm, and matrix size = 256 × 256, yielding 208 sagittal slices with a thickness of 1.0 mm and no interslice gap (Siemens Trio at London and Verio at Paris). For Siemens Allegra at Ulm, parameters were the same except for a slice thickness of 1.1 mm and TE = 2.81 ms, flip angle = 9^o^. Finally for the Philips Achieva at Leiden, acquisition parameters were TR = 7.7 ms, TE = 3.5 ms, FA = 8^o^, FOV = 24 cm, and matrix size = 224 × 224, yielding 164 slices with a thickness of 1.0 mm and no inter‐slice gap. For the diffusion‐weighted scans, 42 unique gradient directions were acquired in the London, Paris, and Leiden sites and 47 directions in Ulm (*b* = 1000 s/mm^2^). Seven images with no diffusion weighting (*b* = 0 s/mm^2^) were acquired for London and Paris, three images with no diffusion weighting were acquired for Ulm, and one image with no diffusion weighting was acquired for Leiden. For further details, see Hobbs et al. (2015).

### 2.8 Data preprocessing

DWI images were first brain extracted with FSL BET and motion‐corrected using eddy in FSL (www.fmrib.ox.ac.uk/fsl) (Andersson & Sotiropoulos, 2016). FSL DTIFIT was used to apply the diffusion tensor. Fractional anisotropy (FA), mean diffusivity (MD), axial diffusivity (AD), and radial diffusivity (RD) maps were then derived. All images were checked visually.

**FIGURE 1:**
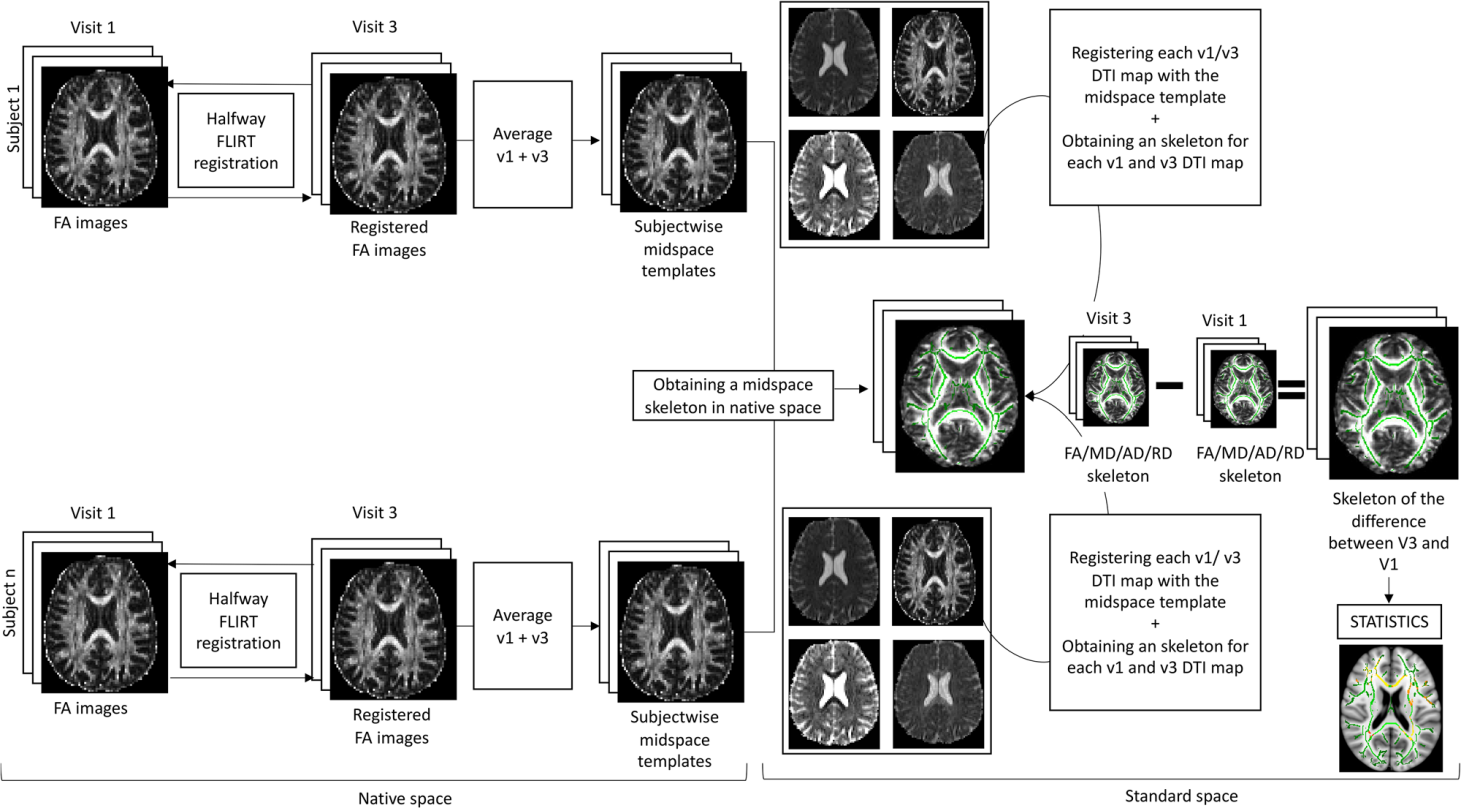
Summary of processing pipeline. Raw FA data from the two visits of all subjects in each study were registered using FSL FLIRT to create halfway images. These images were averaged to create a subject‐wise FA mid‐space template. TBSS automatically aligned all mid‐space images to standard space. The warped FA templates were used to create the mean FA map. This map was thresholded at FA >0.2 to create the midspace FA skeleton for all subjects. All halfway image skeletons from the two visits for all DTI metrics were projected to the standardized FA skeleton. Finally, the skeleton for visit 3 was subtracted from the skeleton for visit 1 and statistical analysis was performed on the subtraction images to compare between HD gene expansion carriers and controls. The same process was repeated to obtain a template only in HD expansion carriers from each study in order to run the correlations in the subtracted images (adapted and reproduced with permission from Engvig et al., 2012). DTI, Diffusion Tensor Imaging; FA, fractional anisotropy; HD, Huntington's disease; TBSS, tract‐based spatial statistics.

We then used an optimized version of the longitudinal pipeline developed by Engvig et al. (Engvig et al., 2012) (Figure 1). In short, for each participant, baseline FA maps were linearly registered to final visit FA maps and vice versa using FSL FLIRT. Both volumes were resampled into the halfway space between the two. Then, the two halfway registered FA maps were averaged to generate a participant midspace FA image. Next, standard TBSS (S. M. Smith et al., 2006) was applied into the midspace images, using the FMRIB_58FA as a target, and images were thresholded at FA >0.2, resulting in the skeleton for the midspace images.

FSL's *tbss_non_FA* was then applied to the baseline and final visit halfway FA maps, using the midspace images as targets. This resulted in the FA skeleton for both the baseline and final study visit in the same space as the midspace skeleton.

Next, baseline and follow‐up images for each non‐FA DTI metric MD, RD, and AD were linearly registered to each other using the transformation parameters from the longitudinal registration of FA images, obtaining halfway maps for each metric in the baseline and follow‐up images. FSL's *tbss_non_FA* was then applied in the halfway AD, MD, and RD maps obtaining a skeleton for each visit's non‐FA DTI metric in the same space as the midspace skeleton.

For each DTI metric, the skeleton from the final visit was subtracted from the skeleton for baseline visit and standard TBSS statistical analysis was performed in the resulting DTI map. Visual inspection of resulting images was performed after each step to check for errors.

The templates and skeletons were created and analyzed in each cohort, grouping together healthy controls and expansion carriers to investigate the differences between HD expansion carriers and controls.

The same process was performed exclusively in HD gene expansion carriers for the evaluation of correlations between clinical scales and structural connectivity.

### 2.9 Statistical analysis

Demographic variables between groups were evaluated using *t*‐tests for mean comparisons between the two groups. One‐way analysis of variance (ANOVA) was used for mean comparisons between more than two groups. Chi‐squared test was used to compare proportions where appropriate. Statistical analysis of demographic variables was performed using Stata v12.0 (StataCorp, College Station, TX, USA).

All analyses were performed using non‐parametric permutation‐based voxel‐wise analysis (*n* = 5000) (Winkler et al., 2014) For all analyses, age, sex, and site were included as covariates. TBSS results are presented cluster‐corrected using Threshold‐Free Cluster Enhancement (TFCE) (*p* < .05) (S. Smith & Nichols, 2009). For group comparisons, we examined change in each diffusion metric over time between controls and HD gene expansion carriers.

TBSS voxel‐wise correlation analyses were performed in the longitudinal DTI maps of the HD gene expansion carrier group only. We examined positive and negative correlations between change in diffusion metrics and baseline scores in disease burden score, SDMT, SWR, TMS, TFC, and the cUHDRS (Schobel et al., 2017).

## 3 RESULTS

Both cohorts had similar mean age in the control groups. Age in the gene‐expansion groups was significantly higher in PADDINGTON, which is consistent with manifest HD participants being on average older than pre‐HD. There were no differences in male/female proportions between controls and expansion‐carriers in either cohort. Mean number of CAG repeats was comparable between both cohorts, while as expected, expansion carriers from TrackOn‐HD had significantly lower disease burden score and lower TMS values than expansion carriers from PADDINGTON.

### 3.1 Group differences

**FIGURE 2:**
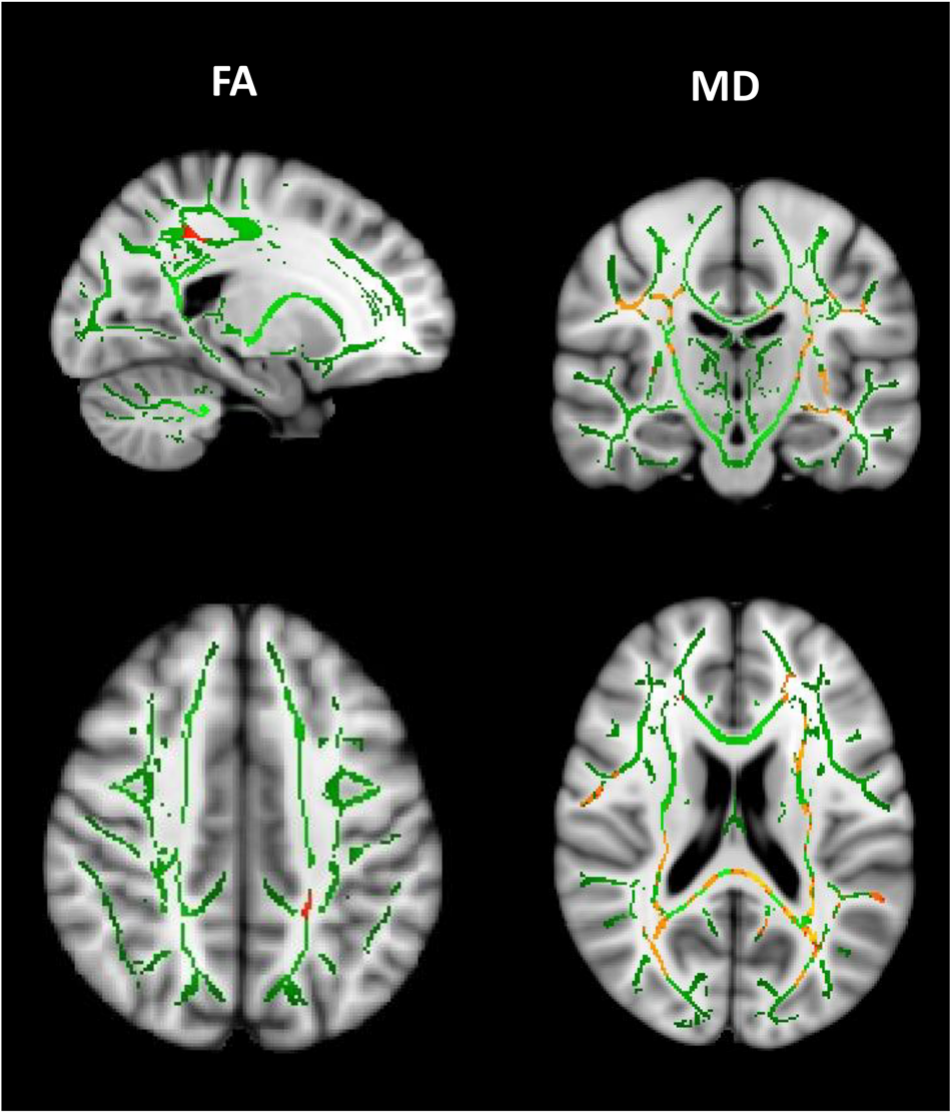
Longitudinal changes in FA and MD between HD expansion‐carriers and controls in the TrackOn‐HD cohort. Data shown are areas with significant decreases in FA or increases in MD in HD expansion‐carriers compared to healthy controls. Results are shown on the FA skeleton (green), overlaid on the MNI standard brain template. All analyses presented are adjusted by age, sex, and study site; thresholded at *p* < .05 (TFCE cluster‐corrected). The color bar (yellow: red, higher: lower) represents *p*‐values above the statistical threshold for significance. FA, fractional anisotropy; HD, Huntington's disease; MD, mean diffusivity; MNI, Montreal Neurological Institute; TBSS, tract‐based spatial statistics; TFCE, threshold‐free cluster enhancement.

In the TrackOn‐HD cohort, expansion‐carriers compared to controls showed significant localized decreases in FA over time in the left superior longitudinal fasciculus when compared to controls (Figure 2). MD was also significantly increased over time in extensive areas including the superior longitudinal fascicles, splenium of the corpus callosum, corona radiata, and external capsules bilaterally in the HD group compared to controls (Figure 2), with both findings indicating longitudinal WM microstructural damage.

There were no significant differences in AD or RD.

**FIGURE 3:**
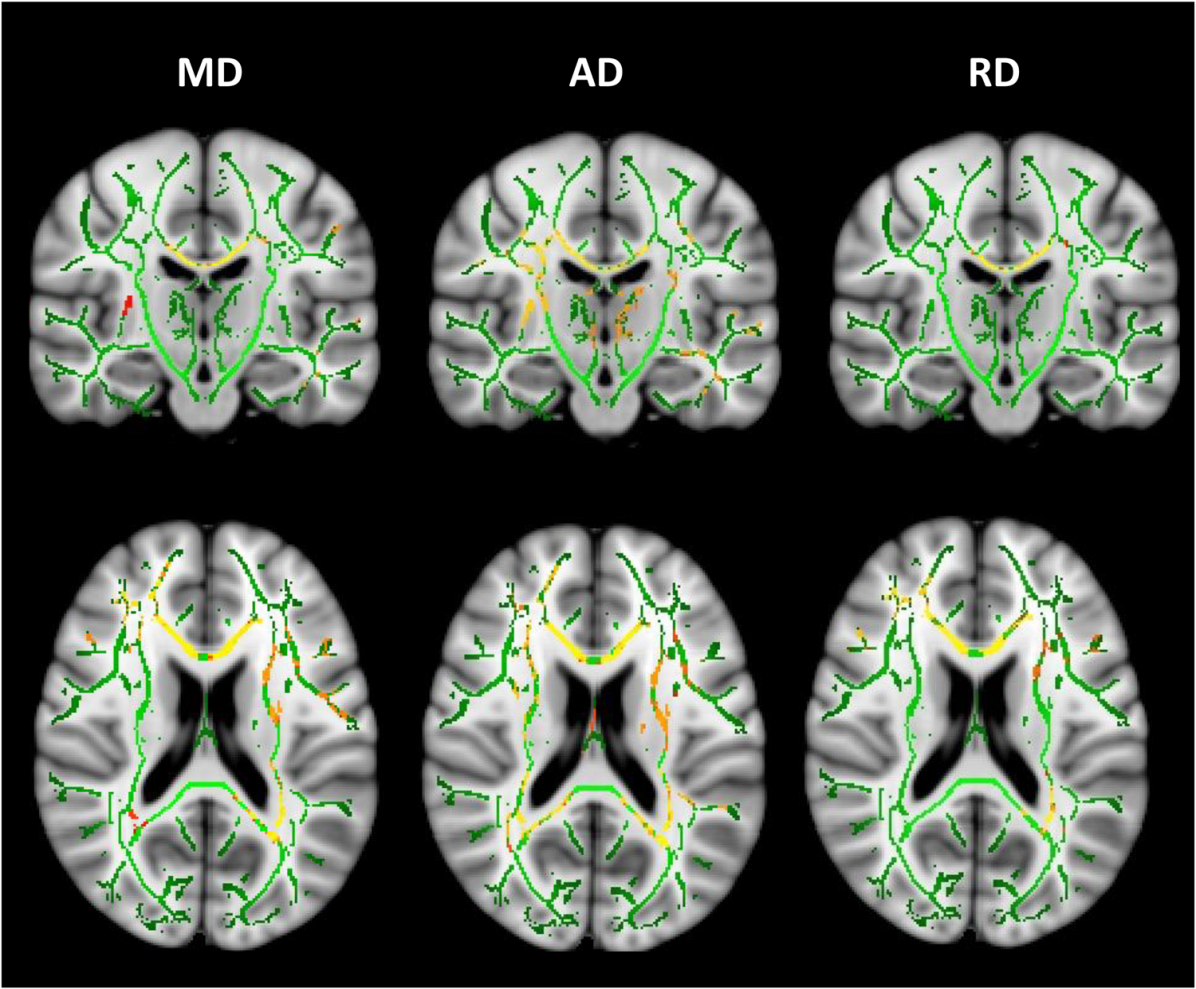
Longitudinal changes in MD, AD, and RD between HD expansion carriers and controls in the PADDINGTON cohort. Data shown are areas with significant increases in MD, AD, and RD in HD expansion‐carriers compared to healthy controls. Results are shown on the FA skeleton (green), overlaid on the MNI standard brain template. All analyses presented are adjusted by age, sex, and study site; thresholded at *p* < .05 (TFCE cluster‐corrected). The color bar (yellow: red, higher: lower) represents *p*‐values above the statistical threshold for significance. AD, axial diffusivity; FA, fractional anisotropy; HD, Huntington's disease; MD, mean diffusivity; RD, radial diffusivity; TBSS, tract‐based spatial statistics; TFCE, threshold‐free cluster enhancement.

For the PADDINGTON cohort, there were longitudinal MD, AD, and RD increases in the corpus callosum and corona radiata, fornix, frontal subcortical WM, and external capsules in expansion‐carriers when compared to controls (Figure 3), indicative of progressive WM disorganization. There were no significant differences in FA between HD expansion‐carriers and controls. There was no evidence of significant longitudinal decreases in MD, AD, or RD in HD expansion‐carriers compared to controls.

### 3.2 Correlations with clinical scales

For the TrackOn‐HD cohort, there was a significant positive correlation between MD, AD, and RD and baseline TMS scores, indicating that larger increases in diffusivity were associated with higher TMS scores (worse motor symptoms) at baseline (Figure S3). Brain regions associated with the TMS involved the corpus callosum and corona radiata bilaterally (Figure S3). There was no correlation between FA and TMS scores. Baseline SWR was negatively correlated with MD in the genu of the corpus callosum and left anterior corona radiata. There were no significant correlations between any DTI metric and disease burden score, SDMT, TFC, or cUHDRS in TrackOn‐HD.

**FIGURE 4:**
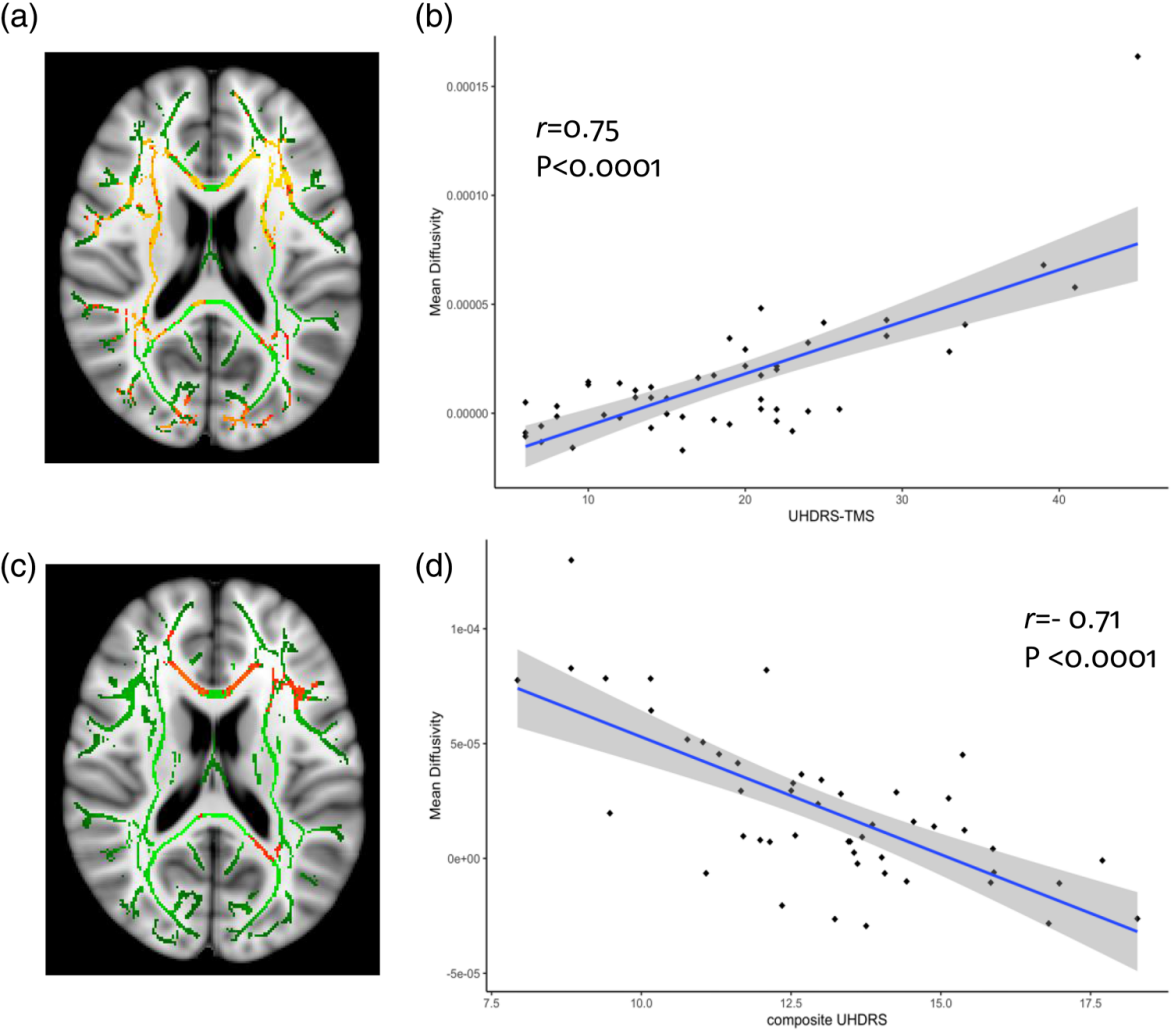
Associations between baseline UHDRS‐TMS and change in mean diffusivity (A and B) and between baseline composite UHDRS and change in mean diffusivity (C and D) in HD expansion‐carriers in the PADDINGTON cohort. (A) TBSS results showing areas with significant positive correlations between change in mean diffusivity and baseline scores in the UHDRS‐TMS. (B) Scatterplot, linear trend, and 95% confidence interval depicting the association between baseline scores in UHDRS‐TMS and change in mean diffusivity within the largest significant cluster from (A). (C) TBSS results showing areas with significant negative correlations between change in mean diffusivity and baseline scores in the composite UHDRS. (D) Scatterplot, linear trend, and 95% confidence interval depicting the association between baseline composite UHDRS and change in mean diffusivity within the largest significant cluster from (C). TBSS results are shown on the FA skeleton (green), overlaid on the MNI standard brain template. All analyses presented are adjusted by age, sex, and study site; thresholded at *p* < .05 (TFCE cluster‐corrected). The color bar (yellow: red, higher: lower) represents *p*‐values above the statistical threshold for significance. HD, Huntington's disease; TBSS, tract‐based spatial statistics; TFCE, threshold‐free cluster enhancement; UHDRS‐TMS, Unified Huntington's Disease Rating Scale—total motor score

For the PADDINGTON cohort, there was a significant, counterintuitive positive correlation between FA in the right posterior corona radiata and baseline TMS scores, indicating that larger decreases in FA were associated with lower TMS scores (less severe motor signs) at baseline in a single cluster of 854 voxels (Figure S4). There were also widespread correlations in the predicted positive direction between baseline TMS scores with MD (Figure 4) and AD (Figure S4) and a positive association between RD and baseline TMS limited to the left anterior corona radiata (Figure S4). Baseline SDMT correlated negatively with MD and AD in the corpus callosum and corona radiata and baseline TFC with AD only in the genu of the corpus callosum, indicating that larger scores in these scales were associated with smaller decreases in structural connectivity.

Similarly, the cUHDRS also correlated negatively with MD and AD in the corpus callosum, corona radiata and subcortical WM tracts (Figure 4). A also suggesting that more severe symptoms at baseline were associated with larger longitudinal increases in diffusivity. There were no correlations between any of the DTI metrics and disease burden score and SWR in PADDINGTON.

## 4 DISCUSSION

In the current study, we have shown robust, longitudinal changes in WM organization across the time course of HD comparing HD expansion‐carriers and controls. Using diffusion MRI, we applied a whole‐brain approach, optimized for longitudinal analysis, to two well‐characterized HD cohorts: TrackOn‐HD, with mainly pre‐HD gene expansion carriers followed over 2 years; and PADDINGTON, with higher disease burden consisting of manifest HD gene expansion carriers investigated over 15 months. TrackOn‐HD revealed longitudinal decreases in FA specific to the superior longitudinal fasciculus, with widespread increases in MD. These WM changes extended to the external capsules, corona radiata, and bilateral subcortical WM in the manifest HD group across all diffusivity measures examined. Our findings suggest a trajectory of progressive WM microstructural disorganization in HD. This is important not only in terms of understanding the mechanisms underlying WM change in HD, but also with a view to diffusion measures acting as biomarkers for drug efficacy and safety in clinical trials.

Evidencing a significant deterioration in a given biomarker with disease progression is essential to show a hypothetical improvement following the administration of a therapy. However, it is challenging to recruit large cohorts of HD participants with available longitudinal DWI data. Among the few HD studies evaluating diffusion metrics longitudinally, most used a region of interest (ROI)‐based approach also showing longitudinal decreases in anisotropy and increases in diffusivity in the corpus callosum of HD patients without significant differences before motor onset (Domínguez et al., 2016; Gregory et al., 2015; Harrington et al., 2016; Sritharan et al., 2010; Weaver et al., 2009). However, ROI studies require selection in advance of the areas being investigated. In contrast, newer drugs targeting *HTT* nucleic acids have different HTT‐lowering potential across brain areas. For example, intrathecally administered antisense oligonucleotides reach maximum concentrations in the cortex with decreased concentrations in deeper brain areas, while microRNAs in clinical development are injected into the striatum with lower concentrations in the cortex and subcortical WM (Tabrizi et al., 2019).

Moreover, mHTT aggregates and neuronal loss do not entirely co‐localize with atrophy, suggesting the interaction of cell autonomous and non‐autonomous factors alongside different protein isoforms, underlying neuronal death (Ast et al., 2018; Hackam, 1999; Raj & Powell, 2021; Ross et al., 2014). Therefore, unbiased whole‐brain imaging analysis can detect alterations in WM microstructure in areas a priori not expected to change.

The main limitations of whole‐brain longitudinal analyses of DWI data are registration failures between baseline and final visit scans leading to misalignment and potential spurious results. However, our use of a midspace template minimizes registration failures. Subsequently, detailed quality control in our analyses showed excellent alignment of baseline and follow‐up DTI maps with the midspace templates.

Importantly, here, we have applied the same longitudinal pipeline to two populations with different disease burden suggesting progressive decreases in structural connectivity as the disease develops. The changes we observed are largely consistent with those previously identified in WM studies for both pre‐HD (Harrington et al., 2016) and manifest HD (Gregory et al., 2015; Poudel et al., 2015; Weaver et al., 2009), in addition to previous pathological findings (Vonsattel et al., 2011). Interestingly, FA decreases over time were present only in the pre‐HD cohort (at our chosen statistical threshold), perhaps indicating that FA may be less sensitive than other DTI metrics to change in manifest HD (Hobbs et al., 2015; Sritharan et al., 2010; Sweidan et al., 2020). These results may be caused by further degeneration of specific WM tracts after clinical motor onset, reducing the complexity of fiber architecture in areas with complex configurations and crossing fibers such as the corona radiata.

FA decreases in TrackOn‐HD were present exclusively in the left hemisphere, while diffusion changes in manifest participants were bilateral and more confluent. Lateralized findings have also been reported in previous HD diffusion studies (McColgan et al., 2017; Odish et al., 2015) suggesting that initially subtle changes in microstructural disorganization may be asymmetric although these tend to coalesce with disease progression. Importantly, these changes expanded toward neighboring areas, becoming bilateral when applying lower statistical thresholds.

To understand the impact of WM microstructural change over time on function and behavior, we correlated baseline clinical scores and change in diffusion measures. Increased pathological burden is a likely explanation for the progressive changes that we identified. We found that TMS was routinely associated with longitudinal changes in WM across both cohorts, indicating increased levels of WM disorganization as motor symptoms worsened. Even for the pre‐HD group, where motor symptoms were less discernable, TMS was a good predictor of WM change. This is consistent with previous studies showing significant relationships between WM microstructure and TMS (Harrington et al., 2016; Hong et al., 2018) with motor scores being able to differentiate pre‐HD from healthy controls (Georgiou‐Karistianis et al., 2013). There were counterintuitive correlations between the TMS and FA in the PADDINGTON cohort suggesting that in this population milder motor symptoms at baseline are associated with larger longitudinal decreases in FA. However, FA was the only DTI metric that did not show significant longitudinal change in PADDINGTON in our analysis, indicating that it may not be particularly sensitive in manifest participants. In addition, the significant area was very localized and not associated with other clinical scales suggesting that the positive correlation between FA and the TMS may be a consequence of longitudinal changes in crossing fibers caused by volumetric decreases. Crossing fibers are not modeled through DTI indices but may be present in 90% of brain voxels, being particularly problematic in areas with complex WM configurations such as the corona radiata, where counterintuitive associations were found in our study (Jeurissen et al., 2013).

Newer advanced multishell diffusion images and myelin‐sensitive modalities such as quantitative magnetization transfer (qMT) imaging have shown myelin impairments from premanifest stages of the disease. Similarly, quantitative susceptibility mapping is sensitive as a myelin marker and could provide further insights into structural connectivity in these areas (Casella et al., 2022, 2020; Heath et al., 2018). In contrast, neurite orientation dispersion and density imaging (NODDI) analysis showed decreased neurite density and increased orientation dispersion in expansion carriers compared to healthy controls (H. Zhang et al., 2012). These techniques could overcome some of the limitations from DTI metrics in future studies investigating longitudinal change in large observational cohorts.

Interestingly, there were no correlations between disease burden score and WM microstructure, indicating that for future studies TMS is a more robust predictor of change (Gregory et al., 2015, 2020; Poudel et al., 2015).

The cUHDRS is a multidomain measure that was generated using data from 1600 early HD participants. It has good test–retest reliability and longitudinal signal‐to‐noise ratio (Schobel et al., 2017). In addition, it is associated with clinically meaningful change (Trundell et al., 2019), and it has been used—alongside the TFC—as the primary outcome measure in GENERATION‐HD1, an HTT‐lowering phase 3 clinical trial (Hoffman‐La Roche, 2019). In the present study, correlations between baseline cUHDRS and WM change overlapped considerably with those tracts in which we identified TMS associations. This would appear to suggest that as a key component of cUHDRS, the TMS may be driving these findings. Although cUHDRS scores have been shown to correlate with measures of WM microstructure at the cross‐sectional level in the TrackHD cohort (Estevez‐Fraga et al., 2021), here we found no such associations in the TrackOn‐HD cohort for longitudinal WM change, being consistent with the lower TMS alongside maximum TFC scores among premanifest participants. However, for manifest HD gene expansion carriers from the PADDINGTON cohort, lower baseline cUHDRS scores did predict increases in diffusivity (Schobel et al., 2017) supporting the use of this score in clinical trials including symptomatic HD participants.

Changes in WM reflect the distribution of pathology from postmortem histological studies, with alterations starting in deep brain areas and progressing toward the cortex during pre‐HD stages (Vonsattel et al., 2011). Therapies that potentially decrease the pathological load of HD, such as mHTT lowering therapies, may also slow WM disorganization, which can be tested in an exploratory fashion as part of a clinical trial. ROI‐based approaches bear sufficient effect sizes to show treatment benefit over a clinical trial period but require a preselection of the structures being analyzed (Hobbs et al., 2015; Rosas et al., 2006; Georgiou‐Karistianis, Scahill et al., 2013).

Here, we have shown that a reproducible, unbiased whole‐brain methodology could detect change over a clinical trial period without the need for an a priori selection of brain regions. In consequence, this methodology could be applied to investigate treatment effects. We considered it important to apply this longitudinal pipeline to investigate the changes in standard DTI metrics, as these have been extensively in cross‐sectional studies (Estevez‐Fraga, Scahill, Rees et al., 2021). However, longitudinal change in newer diffusion metrics such as fixel‐based analysis or NODDI metrics could be explored in future analyses.

A whole‐brain approach is a significant advantage since invasive delivery of newer mHTT lowering therapies will influence the spatial distribution of neuronal changes in response to it. Avoiding preselection of brain areas for investigation could increase the likelihood of detecting significant results without introducing selection bias.

There are some limitations to our study. Although the TrackOn‐HD cohort focused on (and was predominantly composed of) pre‐HD, there was a small proportion of participants after clinical motor onset. However, the distinction between pre‐HD and manifest HD is based on the diagnostic confidence level scale, being highly subjective (Oosterloo et al., 2021). In contrast, newer classifications of HD include objective measures in clinical scores and from imaging biomarkers and are focused on the initial stages of the disease (Tabrizi et al., 2021). In addition, the progression of HD from the imaging perspective is continuous rather than dichotomous (Tabrizi, 2009) The disease burden score differed between both cohorts in our study, with higher disease severity in PADDINGTON. The disease burden score depends on age and CAG repeat length, the main determinants of disease severity and age at onset (Wexler et al., 2004) being therefore more appropriate to estimate the severity of the disease than the proportion of pre‐HD and manifest HD participants. Therefore, our results indicate progressive WM disorganization across the disease time course.

In addition, longitudinal follow‐up was shorter in PADDINGTON (15 months) compared to TrackOn‐HD (24 months). Shorter observation time frames are associated with smaller changes. Therefore, a longer follow‐up period in PADDINGTON would possibly show even more extensive changes, also supporting increasing alterations in diffusion imaging as the disease progresses. A notable proportion of participants in TrackOn‐HD were excluded because of insufficient data at baseline or follow‐up or failed quality control. This could be improved with more rigorous data collection. However, only five participants were excluded due to failures in longitudinal registration, suggesting that this longitudinal pipeline can be applied in further datasets.

Our technical approach sought to minimize misalignment between scans, but there may be minor residual registration errors. However, we performed detailed quality control at each processing stage to avoid such misalignment registration errors. Furthermore, TBSS studies have proven to be reliable and reproducible in HD, differentiating patients from controls and exhibiting progressive WM change in HD (Della Nave et al., 2010; Gregory et al., 2020; J. Zhang et al., 2018).

## 5 CONCLUSION

To conclude, we have shown progressive widespread longitudinal changes in diffusivity and FA in two cohorts with different disease load. These changes parallel the distribution of pathology in HD and are associated with clinical outcomes, including the cUHDRS in manifest participants, being currently used in clinical trials with HTT‐lowering therapies. In consequence, our methodology could be applied in future clinical trials and may be relevant for the development of future therapies directly targeting disease‐causing mechanisms.

## AUTHOR CONTRIBUTIONS

Organization, execution, design, execution, review and critique, writing of the first draft, and review and critique: Carlos Estevez‐Fraga. Organization, execution, design, execution, and review and critique: Michael S. Elmalem. Conception, design, and review and critique: Marina Papoutsi. Review and critique: Alexandra Durr, Elin M. Rees, Nicola Z. Hobbs, Raymund A. C. Roos, Bernhard Landwehrmeyer, Blair R. Leavitt, Douglas R. Langbehn, Rachael I. Scahill, Geraint Rees, and Sarah J. Tabrizi. Conception, organization, execution, design, execution, and review and critique: Sarah Gregory.

## CONFLICT OF INTEREST STATEMENT

During the previous 12 months, Carlos Estevez‐Fraga, Sarah Gregory, Rachael I. Scahill, Geraint Rees, and Sarah J. Tabrizi report support from a Wellcome Trust Collaborative Award (200181/Z/15/Z).

Sarah J. Tabrizi receives research grant funding from the CHDI Foundation, Vertex Pharmaceuticals, the UK Medical Research Council, the Wellcome Trust (200181/Z/15/Z), and the UK Dementia Research Institute that receives its funding from DRI Ltd., funded by the UK MRC, Alzheimer's Society, and Alzheimer's Research UK. She has undertaken consultancy services for Alnylam Pharmaceuticals Inc., Atalanta Pharmaceuticals (SAB), F. Hoffmann‐La Roche Ltd./Genentech, Guidepoint, Horama, Locanobio, LoQus23 Therapeutics Ltd. (SAB), Novartis Pharma, PTC Therapeutics, Sanofi, Spark Therapeutics, Takeda Pharmaceuticals Ltd., Triplet Therapeutics (SAB), University College Irvine, Vertex Pharmaceuticals Incorporated, and Wave Life Sciences. All honoraria for these consultancies were paid through the offices of UCL Consultants Ltd., a wholly owned subsidiary of University College London. Sarah J. Tabrizi has a patent Application number 2105484.6 on the FAN1‐MLH1 interaction and structural analogs licensed to Adrestia Therapeutics.

Douglas R. Langbehn receives academic research funding from CHDI, NINDs, the University College of London (UCL), and the Wellcome Trust via (UCL). He reports personal consulting fees and non‐financial support from Voyager Therapeutics, personal consulting fees from Novartis, personal consulting fees and non‐financial support from uniQure, personal consulting fees from Takeda, personal consulting fees from AskBio, and personal consulting fees from Spark Therapeutics, all outside the submitted work.

Bernhard Landwehrmeyer has provided consulting services, advisory board functions, clinical trial services, and/or lectures for Acadia Pharmaceuticals, Affiris, Allergan, Alnylam, Amarin, AOP Orphan Pharmaceuticals AG, Bayer Pharma AG, Boehringer‐Ingelheim, CHDI Foundation, Deutsche Huntington‐Hilfe, Desitin, Genentech, Genzyme, GlaxoSmithKline, F. Hoffmann‐La Roche, Ipsen, ISIS Pharma (IONIS), Lilly, Lundbeck, Medesis, Medivation, Medtronic, NeuraMetrix, Neurosearch Inc., Novartis, Pfizer, Prana Biotechnology, Prilenia, PTC Therapeutics, Raptor, Remix Therapeutics, Rhône‐Poulenc Rorer, Roche Pharma AG Deutschland, Sage Therapeutics, Sanofi‐Aventis, Sangamo/Shire, Siena Biotech, Takeda, Temmler Pharma GmbH, Teva, Triplet TX, Trophos, UniQure, and Wave Life Sciences. He has received research grant support from the CHDI Foundation, the Bundesministerium für Bildung und Forschung (BMBF), the Deutsche Forschungsgemeinschaft (DFG), the European Commission (EU‐FP7), EU Joint Programme—Neurodegenerative Disease Research (JNPD), and ERA‐Net for Research Programmes on Rare Diseases (E‐Rare). His study site has received compensation in the context of the observational REGISTRY‐Study of European Huntington's Disease Network (EHDN) and the global observational Enroll‐HD. In the context of clinical trials, his institution, the University Hospital of Ulm, has received compensation from Allergan, Ionis, F. Hoffmann‐La Roche, Pfizer, and Teva.

Alexandra Durr serves on scientific advisory boards for Triplet Therapeutics and receives laboratory funding from BIOGEN, all outside the submitted work.

Blair R. Leavitt is on the Scientific Advisory Board of sRNAlytics (GateHouse Bio) for which he received stock options, and reports scientific consultancy fees from Teva, Roche/Genentech, Takeda, Triplet, Ionis, Novartis, Spark, Scintetica, LifeEdit, Design, Remix Therapeutics, and PTC Therapeutics. Dr Leavitt's Laboratory has obtained previous and current research grants from CIHR, HSC, NMIN, CHDI, Teva, ProMIS and uniQure. He is a founding co‐Editor‐in‐Chief, Journal of Huntington's Disease, Former Co‐Chair of the Huntington Study Group, and is a Co‐Founder and CEO of Incisive Genetics Inc., in which he has stock and stock options. Incisive Genetics Inc. is an early‐stage pre‐clinical biotechnology company that was founded to develop in vivo lipid nanoparticle delivery of CRISPR/Cas9 genome editing. This is not a therapeutic approach that is currently in clinical testing for HD, nor is this approach in late pre‐clinical stages. The company has no products to endorse, does not have an IND for HD, nor are any commercial efforts currently underway. Sarah J. Tabrizi was the global principal investigator (PI) for TrackOn‐HD. Alexandra Durr, Blair R. Leavitt, Raymund A. C. Roos, and Bernhard Landwehrmeyer were site PIs for Paris, Vancouver, Leiden, and Ulm, respectively. No other relevant disclosures or conflicts of interest.

Michael S Elmalem, Nicola Z. Hobbs, and Elin M. Rees declare no conflict of interest.

### PEER REVIEW

The peer review history for this article is available at https://publons.com/publon/10.1002/brb3.2940.

## Supporting information

Figure S1 ‐ Flow diagram Track‐On HD cohort

Figure S2 ‐ Flow diagram PADDINGTON cohort

Figure S3 ‐ Statistically significant associations between change diffusivity metrics and baseline scores in TMS in HD expansion carriers in Track‐On HD.

Figure S4 ‐ Statistically significant associations between change in FA, MD, AD and with RD and baseline scores in TMS in HD expansion carriers in PADDINGTON.

## Data Availability

The data that support the findings of this study and the analysis code will be made available upon reasonable request.
