# Supplementary figures and images for: Progressive alterations in white matter microstructure across the timecourse of Huntington's disease
Source: Brain Behav. 2023 Mar 14;13(4):e2940. doi: 10.1002/brb3.2940 (PMC10097137; doi:10.1002/brb3.2940)

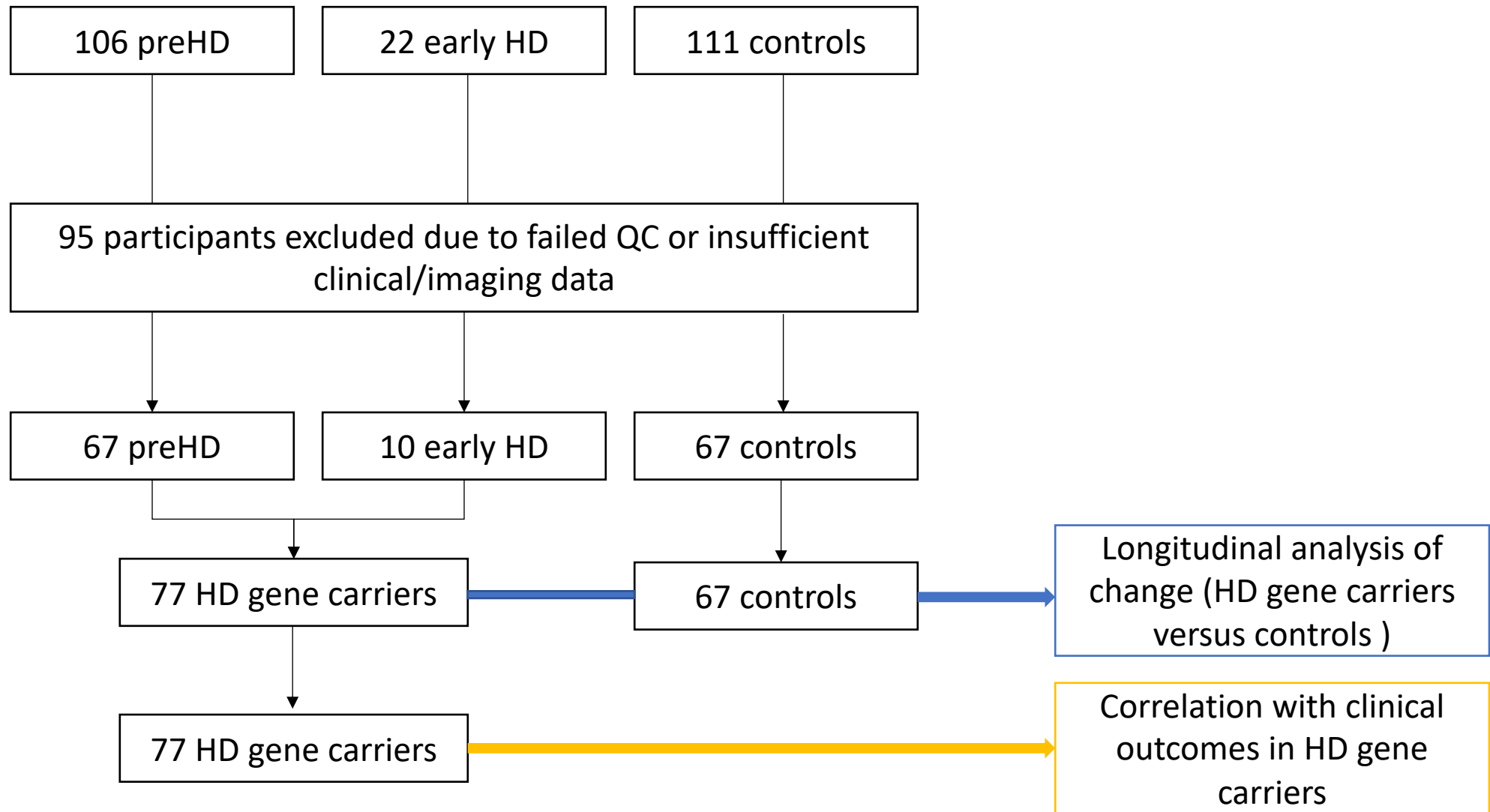

Supplement: Supplementary file 1 — Figure S1 ‐ Flow diagram Track‐On HD cohort [file BRB3-13-e2940-s004.pdf]

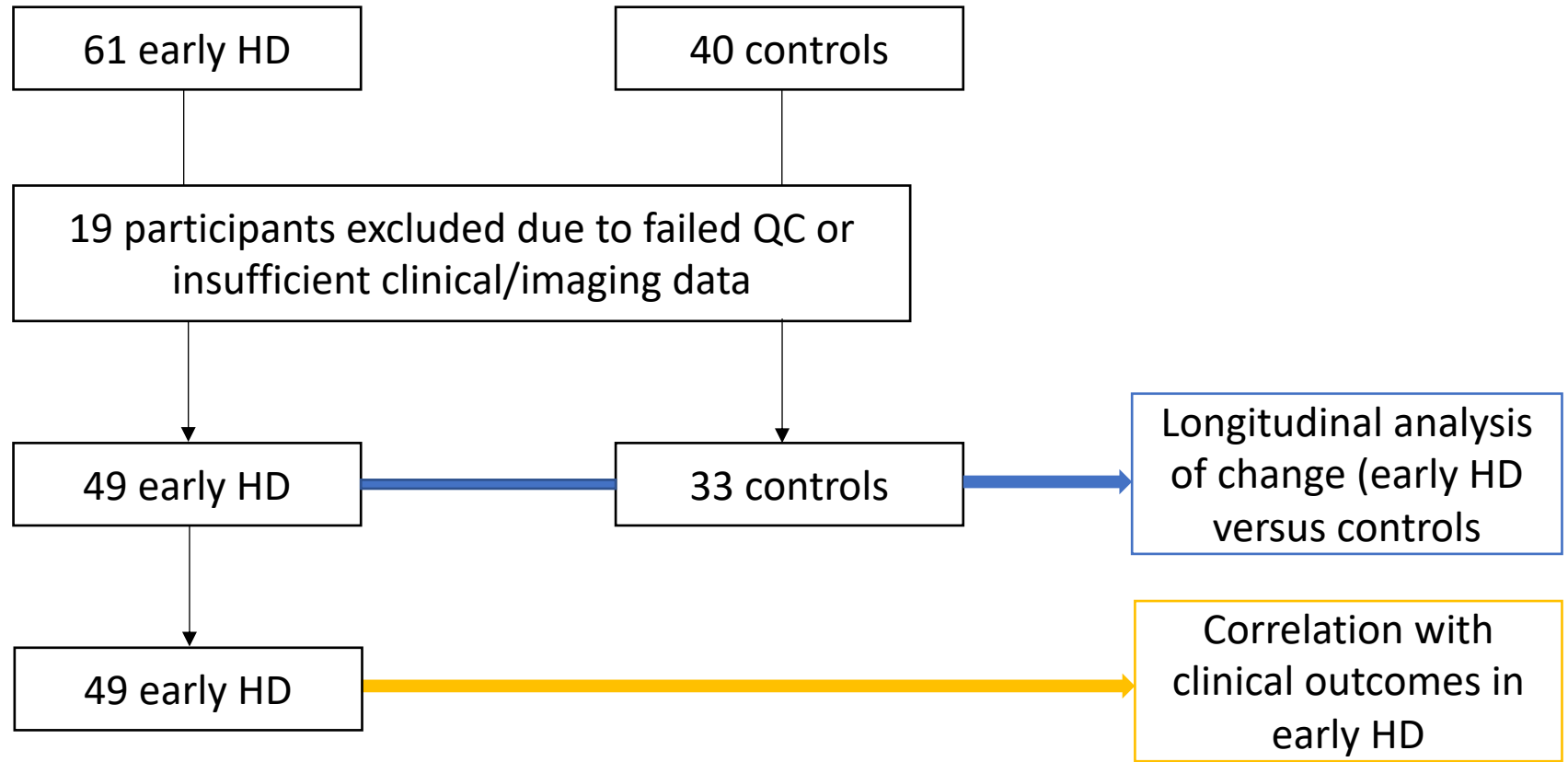

Supplement: Supplementary file 2 — Figure S2 ‐ Flow diagram PADDINGTON cohort [file BRB3-13-e2940-s003.pdf]

MD

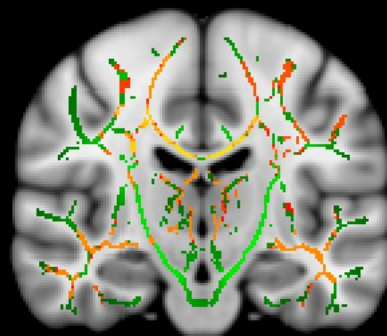

AD

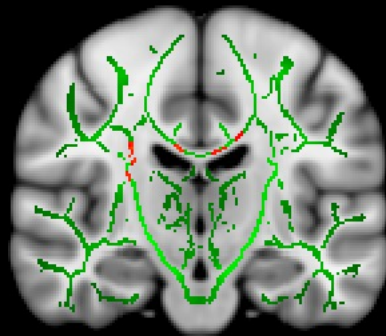

RD

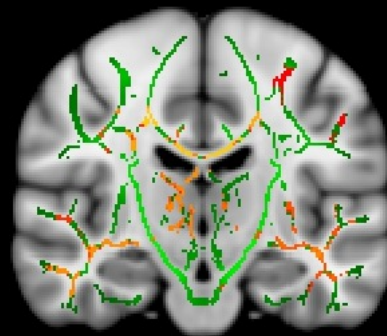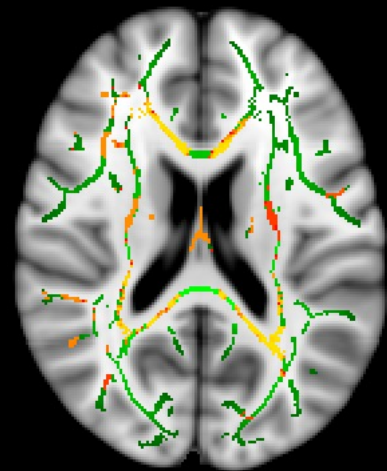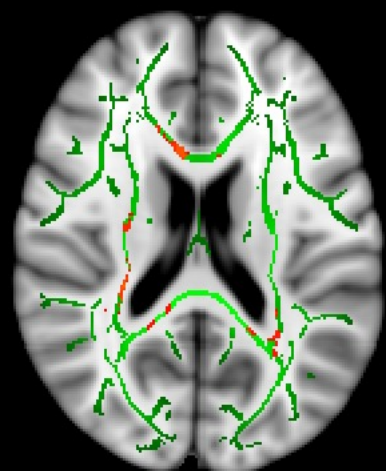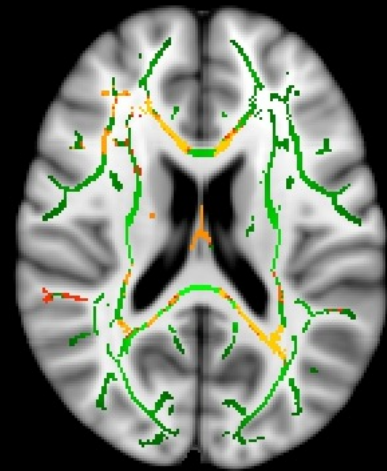

Supplement: Supplementary file 3 — Figure S3 ‐ Statistically significant associations between change diffusivity metrics and baseline scores in TMS in HD expansion carriers in Track‐On HD. [file BRB3-13-e2940-s001.pdf]

FA

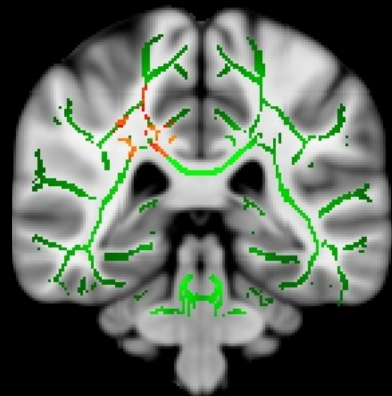

MD

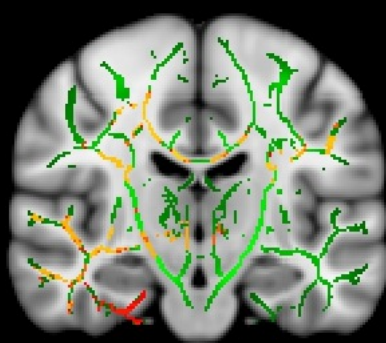

AD

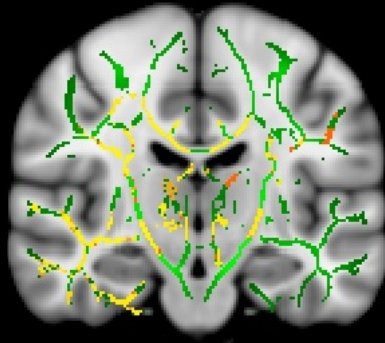

RD

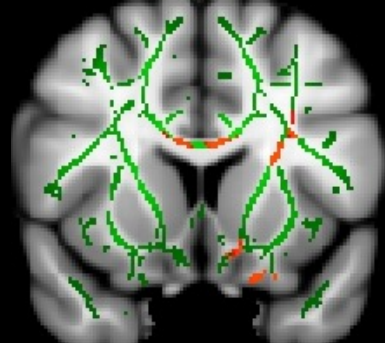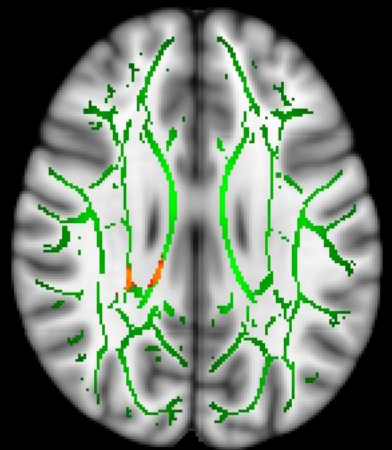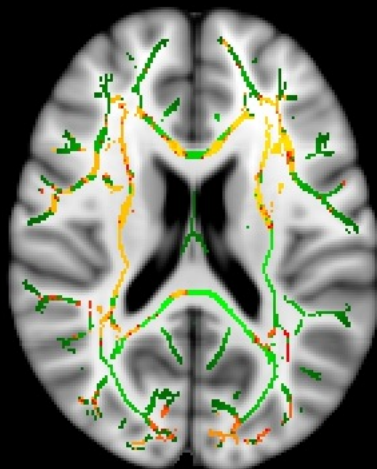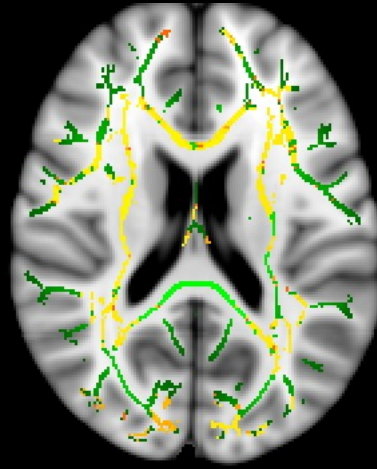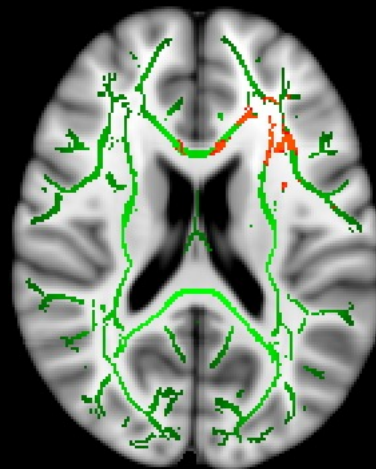

Supplement: Supplementary file 4 — Figure S4 ‐ Statistically significant associations between change in FA, MD, AD and with RD and baseline scores in TMS in HD expansion carriers in PADDINGTON. [file BRB3-13-e2940-s002.pdf]
